# Editorial: Anti-inflammatory drug development focusing on immune mediated diseases

**DOI:** 10.3389/fphar.2024.1413141

**Published:** 2024-05-17

**Authors:** José Fernando Oliveira-Costa, Amit Prasad

**Affiliations:** ^1^ Center for Infusions and Specialized Medicines of Bahia—CIMEB, Bahia State Health Department, Salvador, Brazil; ^2^ School of Biosciences and Bioengineering, Indian Institute of Technology, Mandi, India; ^3^ Center for Indian Knowledge System and Mental Health, Indian Institute of Technology, Mand, India; ^4^ Center for Human Computer Interaction, Indian Institute of Technology, Mandi, India

**Keywords:** inflammation, drugs, therapeutics, molecular docking, repurposing

Inflammatory diseases are distributed worldwide, affecting approximately 5% of the world population (Bieber et al., 2023). Among these the inflammatory diseases like rheumatoid arthritis, type 1 diabetes mellitus, systemic lupus erythematosus, multiple sclerosis, Crohn’s disease, ulcerative colitis, psoriasis, and myasthenia gravis are prominent ones. The ever-increasing number of senior citizens (age >60 years) across the globe and the advent of newer and better diagnostic tools for immune-mediated diseases has produced an increase in the incidence of inflammatory diseases. While the general population grew from one billion in the year 1800 to eight billion at present, and it is estimated that by 2030 one in six people in world will be aged sixty or older (Bolkan et al., 2023; Pereira et al., 2023). In addition to this, studies suggest that there is an increased risk of developing immune-mediated diseases to the individuals who got SARS-CoV-2 infections (Kim et al., 2024). Despite the great advances in the treatment of these medical conditions in recent past, many patients suffer with serious conditions of the disease for a long time. This situation demands constant research, to identify new drugs, with more specific targets and fewer side effects to treat inflammatory diseases (Wang et al., 2015).

In Brazil, despite the guarantee of free access to medicines through the Unified Health System for all citizens as a constitutional right, immune-mediated diseases represent a significant challenge. This is due to its complexity and the lack of adequate resources for early diagnosis and effective treatment. For example, one of the main obstacles to the accurate diagnosis of rheumatological diseases in Brazil is the shortage of specialized professionals. Recent data indicates that 53.3% of the all the rheumatologists in the country live in the capitals of state in the southeast region; 16.8% live in the very populous northeast region, while only 4.1% of medical professionals reside in the north region. This data represents the heterogeneity in the territorial distribution of professionals in Brazil, a developing country. Furthermore, the lack of adequate medical training for those who work in primary care can lead to diagnostic errors or delays in referral to specialists, which ends up worsening the health condition, with irreversible damage to patients (BRAZIL, 2021; Scheffer et al., 2023). The same kind of situation is prevalent in almost all the developing or underdeveloped countries where most of the advance medical facilities are concentrated to affluent and populous cities and rural areas are devoid of such facilities. Despite the existence of specific clinical protocols for treatment, there is a great difficulty in the diagnosis for immune disease due to complex definition of diagnostic criteria, leading to severe impact on the quality of life of affected individuals (Lin et al., 2023). In this scenario, the discovery of new molecules capable of treating diseases mediated by the immune system remains fundamental.

Identification of new natural products, synthetic molecules, drug repurposing, *in silico* molecular modelling studies associated with chemical synthesis represent important strategies for the development of new drugs (Spitschak et al., 2022; Wainwright et al., 2022). The global demand for new drugs and the associated scientific challenges were motivations for this special edition, with the aim to bring together scientific research works that addressed the development of new immunomodulatory drugs. As a result, we have very interesting articles, coming from different research aspects, which lead us to believe that they will be very useful in discovering new treatments for inflammatory diseases in near future.

The study Li et al. investigated adverse events associated with mepolizumab in the treatment of severe asthma with an eosinophilic phenotype. Reports of expected adverse events were identified, but also unexpected ones, such as coughing, malaise and chest discomfort, recommending continued attention to safety in long-term use and in paediatrics.

Other study, entitled Arjsri et al. examined the potential of exiguaflavanone A and B, present in the root of *Sophora exigua*, in suppressing the proliferation and metastasis of non-small cell lung cancer cells. This kind of lung cancer is induced by inflammation. Through inhibition of the NLRP3 inflammasome pathway, these compounds have demonstrated efficacy in reducing the inflammatory response associated with lung cancer.

The article “Dasatinib suppresses particulate-induced pyroptosis and acute lung inflammation " highlights the efficacy of dasatinib in mitigating inflammatory responses triggered by particulate exposure. Through its action on Src family kinases, dasatinib effectively inhibits pyroptosis induced by particulate materials, such as silica particles, while sparing non-particulate-induced pyroptosis. Furthermore, Dasatinib attenuates acute lung inflammation induced due to exposure to particulate by improving phagolysosomal function and reducing inflammatory mediator release.

Interestingly, the authors of Kim et al., investigated the therapeutic potential of N-benzyl-N-methyldecan-1-amine and its derivative in mitigating 2,4-dinitrobenzenesulfonic acid-induced colitis and collagen-induced rheumatoid arthritis. These compounds were evaluated for their efficacy in ameliorating associated inflammatory symptoms and had shown promising anti-inflammatory properties. This further suggests that they should be considered as potential therapeutic agents for colitis and rheumatoid arthritis.

In the study titled Lu et al., authors describe the abundance of natural flavones found in both edible and medicinal plants, presenting a promising avenue for the treatment of ulcerative colitis, highlighting the importance of concentrated detailed effort in exploring natural remedies for several inflammatory gastrointestinal disorders.

The authors of the research article titled Peng et al. explored the development of a sustained-release phospholipid-based phase separation gel containing berberine for the treatment of rheumatoid arthritis. The formulation aims to enhance drug retention at the site of inflammation, thereby improving therapeutic efficacy while minimizing side effects.

In psoriasis, dysregulated Th17 cell responses contribute to skin inflammation and is a major contributor of immune pathologies to the disease. Authors in Lee et al. investigated the therapeutic potential of aminooxy acetic acid (AOA), a known inhibitor of serine metabolism, in alleviating Th17 mediated psoriasis-like skin inflammation. Through *in vitro* and *in vivo* experiments, it is demonstrated that AOA effectively attenuates Th17 cell differentiation and function, leading to reduced production of pro-inflammatory cytokines such as interleukin-17 and interleukin-22. Their findings highlight AOA as a promising therapeutic candidate for the treatment of psoriasis.

The study Xu et al. explores the structural properties and anti-dermatitis effects of flavonoids-loaded gold nanoparticles (AuNPs), using extracts from *Eupatorium japonicum*. The results demonstrate promising structural attributes of the flavonoids-loaded AuNPs, with significant anti-dermatitis effects by the suppression of the production of inflammatory cytokines (RANTES, TARC, CTACK, IL-6, and IL-8) and intracellular reactive oxygen species.

Neostigmine, a well-known acetylcholinesterase inhibitor, has traditionally been utilized for its ability to enhance skeletal muscle strength, particularly in conditions such as myasthenia gravis and neuromuscular blockade reversal. The authors of the review article Si et al. had reviewed the past 20 years of research and provided new insight about the role of neostigmine use in clinical application.

The study Zhou et al. looked for the impact of dexmedetomidine (DEX), a norepinephrine release inhibitor, on stroke-associated pneumonia (SAP) in mice model. Despite its role in inhibiting norepinephrine release, DEX did not show any improvement in SAP in the mouse model. This suggests that DEX should be used cautiously to alleviate SAP associated symptoms and further studies are required to fully understand the role of DEX in SAP.

Interstitial lung disease (ILD) encompasses a diverse group of chronic lung disorders characterized by inflammation and fibrosis of the interstitial lung tissue, leading to progressive impairment of lung function. Authors in the review article Huo et al. explore the advancements in therapy and treatment options for ILD, which unfortunately remain limited. One promising avenue involves resveratrol, a natural polyphenolic compound renowned for its diverse pharmacological properties. These include antioxidative, anti-inflammatory, and anti-fibrotic effects. Resveratrol achieves this by inhibiting key signalling pathways such as TGF-β/Smad2/3/4, NF-κB, and JAK/STAT, thereby preventing overactivation of immune cells."

Considering the potential activities of resveratrol, the another study “Resveratrol attenuates staphylococcal enterotoxin B-activated immune cell metabolism via upregulation of miR-100 and suppression of mTOR signalling pathway” investigates the effects of resveratrol on immune cell metabolism activated by staphylococcal enterotoxin B (SEB) and explores the underlying molecular mechanisms showing that resveratrol attenuates SEB-induced immune cell metabolism by upregulating miR-100 and suppressing the mTOR signalling pathway.

In the study Sun et al., authors established a HTS model for identifying compounds capable of inhibiting cell adhesion. Thus, using LPS-induced human umbilical vein endothelial cells (HUVECs) and calcein-AM-labelled human monocytic cell THP-1, they established a HTS model for cell adhesion inhibitors, proving to be suitable for screening and validating cell adhesion inhibitors.

In conclusion, the field of anti-inflammatory drug development for immune mediated diseases is evolving rapidly and breath-taking research, supported by innovative techniques is giving transformative breakthroughs and cutting-edge solutions.

However, despite the current research diversity with this edition and optimism for the future, inflammatory diseases are known for their complexity and will remain an intriguing challenge for researchers for future studies too.
